# Ischemic and hemorrhagic brain injury during venoarterial-extracorporeal membrane oxygenation

**DOI:** 10.1186/s13613-018-0475-6

**Published:** 2018-12-20

**Authors:** Loïc Le Guennec, Clémentine Cholet, Florent Huang, Matthieu Schmidt, Nicolas Bréchot, Guillaume Hékimian, Sébastien Besset, Guillaume Lebreton, Ania Nieszkowska, Pascal Leprince, Alain Combes, Charles-Edouard Luyt

**Affiliations:** 1 Service de Réanimation, Institut de Cardiologie, Groupe Hospitalier Pitié–Salpêtrière, Assistance Publique–Hôpitaux de Paris, 47, boulevard de l’Hôpital, 75013 Paris, France; 2 UMRS_1166-ICAN Institute of Cardiometabolism and Nutrition, Sorbonne Universités, UPMC Université Paris 06, INSERM, Paris, France; 3 Service de Chirurgie Thoracique et Cardiovasculaire, Institut de Cardiologie, Groupe Hospitalier Pitié–Salpêtrière, Assistance Publique–Hôpitaux de Paris, Paris, France

**Keywords:** Ischemic stroke, Brain hemorrhage, Refractory cardiogenic shock, Venoarterial-extracorporeal membrane oxygenation, Carbon dioxide, Blood coagulation disorders

## Abstract

**Background:**

Structural neurological complications (ischemic stroke and intracranial bleeding) and their risk factors in patients receiving venoarterial-extracorporeal membrane oxygenation (VA-ECMO) are poorly described. Our objective was to describe frequencies, outcomes and risk factors for neurological complications (ischemic stroke and intracranial bleeding) in patients receiving VA-ECMO.

**Methods:**

Retrospective observational study conducted, from 2006 to 2014, in a tertiary referral center on patients who developed a neurological complication(s) on VA-ECMO.

**Results:**

Among 878 VA-ECMO-treated patients, 65 (7.4%) developed an ECMO-related brain injury: 42 (5.3%) ischemic strokes and 20 (2.8%) intracranial bleeding, occurring after a median [25th;75th percentile] of 11 [6;18] and 5 [2;9] days of support, respectively. Intracranial bleeding but not ischemic stroke was associated with higher mortality. Multivariable analysis retained only platelet level > 350 giga/L as being associated with ischemic stroke. Female sex, central VA-ECMO and platelets < 100 giga/L at ECMO start were independently associated with intracranial bleeding with respective odds ratios [95% CI] of 2.9 [1.1–7.5], 3.8 [1.1–10.2] and 3.7 [1.4–9.7]. In a nested case–control study, rapid CO_2_-level change from before-to-after ECMO start also seemed to be associated with intracranial bleeding.

**Conclusions:**

Neurological events are frequent in VA-ECMO-treated patients. Ischemic stroke is the most frequent, occurs after 1 week on ECMO support, has no specific risk factor and is not associated with higher mortality. Intracranial bleeding occurs earlier and is associated with female sex, central VA-ECMO, low platelet count and rapid CO_2_ change at ECMO start, and high mortality.

**Level of evidence:**

This study provides Class IV evidence that central VA-ECMO, low platelet count and rapid CO_2_ change at ECMO start are associated with intracranial bleeding and high mortality.

**Electronic supplementary material:**

The online version of this article (10.1186/s13613-018-0475-6) contains supplementary material, which is available to authorized users.

## Introduction

Use of venoarterial-extracorporeal membrane oxygenation (VA-ECMO) to treat refractory cardiogenic shock has increased over the past decade [1–3]. Among complications occurring in ECMO-treated patients, brain injury is among the most frequent, affecting 8–50% [4–10]. These differences across studies are mainly attributable to different brain-injury definitions, with some authors considering only intracranial bleeding, whiles others chose broader definitions. Moreover, risk factors for brain injury and specific lesions (i.e., intracranial bleeding and ischemic stroke) are poorly described. Pertinently, some risk factors may be controllable: a recent retrospective study on venovenous-(VV-)ECMO showed that rapid PaCO_2_-level change at ECMO start was associated with intracranial bleeding [11]. If such controllable brain-injury risk factors in VA-ECMO patients exist, they could impact patients’ outcomes.

Thus, we undertook this retrospective study to describe the frequencies, morbidities and mortalities of structural brain injuries (namely ischemic stroke and intracranial hemorrhage) occurring on VA-ECMO, and attempt to identify their associated risk factors.

## Materials and methods

All patients admitted to our intensive care unit (ICU) over 8 years (2006–2014) who received VA-ECMO support were included. Information on medical history, clinical and biological parameters at ICU admission and during ICU stay was collected prospectively. In particular, any events occurring on ECMO were prospectively recorded in the ICU’s database. The charts of all VA-ECMO-treated patients were retrospectively reviewed and analyzed to identify those with neurological complications.

### Definitions

A clinical neurological complication was defined as any clinical event occurring on ECMO support, including any clinical sign suggestive of stroke (hemiplegia, mydriasis, anisocoria, asymmetry on physical examination), but also confusion, delirium, seizures, coma despite sedation withdrawal. Patients were categorized according to brain-injury presence or absence on cerebral computed-tomography images (i.e., no damage, ischemic stroke, intracranial bleeding), and groups were compared [11]. Patients with hemorrhagic transformation of ischemic stroke were arbitrarily classified as ischemic stroke: Because the first injury is ischemic stroke, we assumed the risk factors associated with hemorrhagic transformation of ischemic stroke were the same than those of ischemic stroke, rather than risk factors for intracranial bleeding. Neurological events occurring pre-ECMO or 7 days post-ECMO removal were not considered as having occurred on ECMO, and those patients were classed as having no damage. Events occurring within 7 days post-ECMO were arbitrarily considered to be on-ECMO brain injuries because a neurological event is sometimes diagnosed only several days after its occurrence, mainly because patients are sedated, making their evaluation difficult. Patients with diffuse microbleeds were not included because this particular condition’s pathophysiology differs from that of ischemic stroke or intracranial bleeding [12]. Central VA-ECMO refers to cannulation performed within atria and ventricles during open-chest surgery, and peripheral VA-ECMO refers to cannulation performed within femoral vessels, either percutaneously or after surgical cut-down.

### Patient management under ECMO

Anticoagulation protocol [11, 13, 14], and membrane oxygenator and its circuitry management are reported in the Additional file 1.

Patients underwent daily routine neurological examinations by ICU physicians and nurses at least once daily after sedation withdrawal, including Glasgow coma scale calculation, response to verbal orders or pain, tendon reflexes, brainstem reflexes and plantar reflex, eye opening and pupil examination, with pupil sizes and their light reactivity assessed every 4 h. Moreover, any unexpected event (e.g., seizures, delirium confusion, no awakening after sedation withdrawal…) was recorded in the medical chart. Once a neurological symptom was observed (including but not restricted to change in neurological examination findings, mydriasis, anisocoria, seizures, delirium, confusion, coma despite sedation withdrawal…), a cerebral computed-tomography scan was obtained within 6 h.

### Statistical analysis

Data are expressed as medians [25th;75th percentile] or means [± standard deviation (SD)], as appropriate. Between-group comparisons were analyzed using Student’s *t* test, the Mann–Whitney *U*-test or Kruskal–Wallis test for continuous variables and Chi-square test for categorical variables. A logistic-regression model was used to test the univariable association of patients’ clinical characteristics and ICU events with the development of intracranial bleeding or ischemic stroke. Thereafter, multivariable logistic-regression models using backward-stepwise variable elimination (with the variable-exit threshold set at *P *> 0.05) compared the factors that were significant in the univariable analyses (*P* ≤ 0.10), and those previously reported to be strongly associated with intracranial bleeding or ischemic stroke were entered into each model. Interactions were tested in the models; variables strongly associated with other(s) were not included in the multivariable model. For univariable and multivariable analyses, continuous variables were dichotomized according to their median values, except for those suspected of being associated with ischemic stroke or intracranial bleeding. To analyze factors associated with ischemic stroke, the following platelet count, fibrinogen and prothrombin time cutoffs at ECMO onset were retained: 350 giga/L, 6 g/L and 70%, respectively, rather than their median values, assuming that patients with “normal” or “supranormal” coagulation parameters could be more at risk of ischemic stroke than patients without. To analyze factors associated with intracranial bleeding, the following platelet count, fibrinogen, aPTT and prothrombin time cutoffs at ECMO start were chosen: 100 giga/L, 1.5 g/L, 3% and 30%, respectively, rather than their median values, postulating that patients with impaired coagulation parameters could be more at risk of intracranial bleeding than patients without.

Because there may be competing risks between the risk of developing neurological complications on ECMO and death, we performed 3 supplementary multivariable analyses: the first one to investigate factors associated with hospital mortality; the second to investigate factors associated with a composite endpoint of death and ischemic stroke; and the third one to investigate factors associated with a composite endpoint of death and intracranial bleeding.

### Nested case–control studies

Two nested case–control studies were designed to explore the role of specific risk factors in intracranial bleeding or ischemic stroke. Controls were patients with no damage matched for age ± 5 years, SAPS II ± 5, ENCOURAGE mortality-risk score ± 5 and ECMO duration. See the Additional file 1 for their methodological details.

Analyses were computed with StatView v5.0 (SAS Institute Inc, Cary, NC) and SPSS v11.5 (SPSS Inc, Chicago, IL) software. *P *< 0.05 defined significance.

## Results

**Fig. 1:**
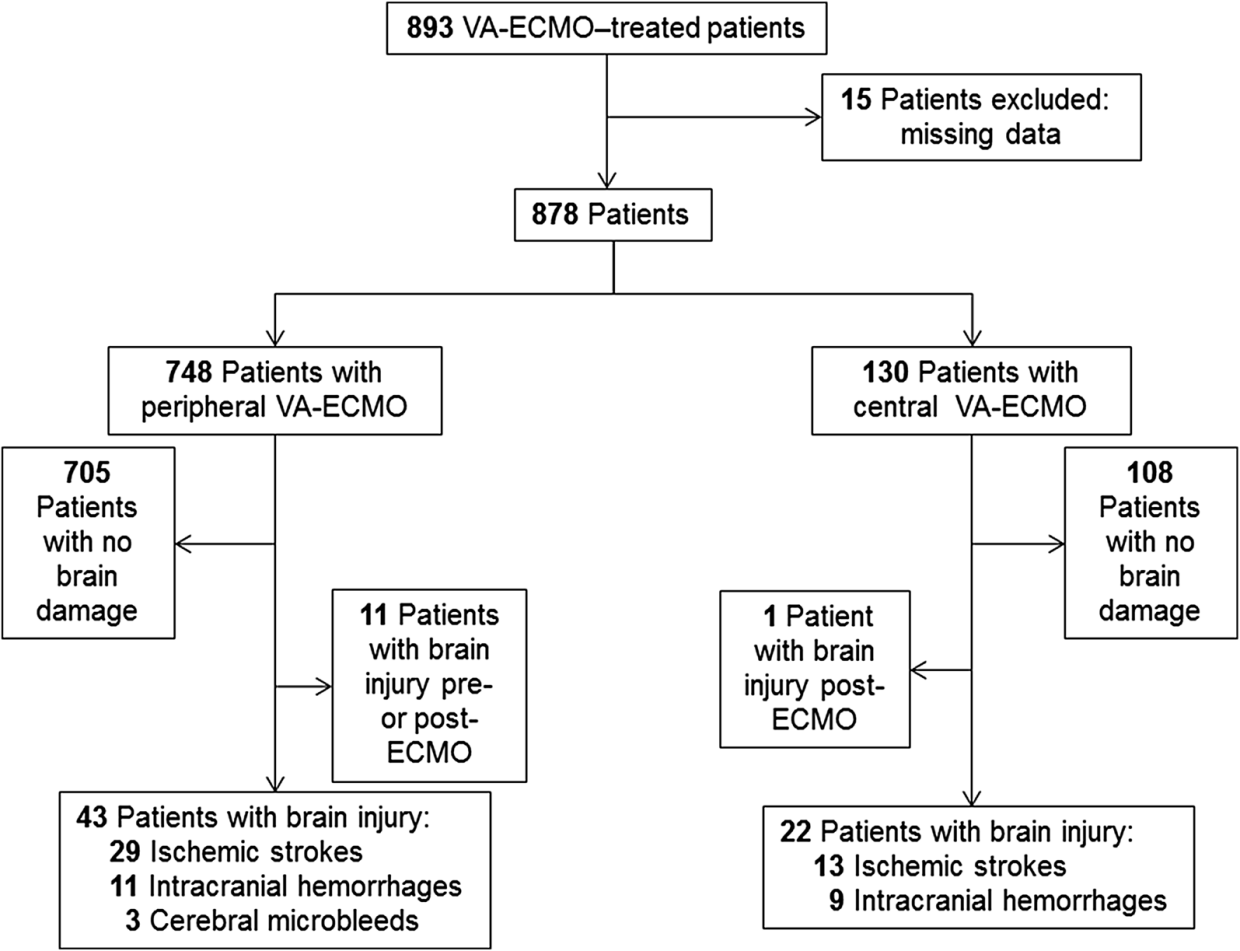
Flowchart of the study. VA-ECMO, venoarterial-extracorporeal membrane oxygenation

During the study period, among the 893 patients requiring VA-ECMO support (Fig. 1), 15 were excluded due to missing data. Among the remaining 878 patients retained for the analysis, 65 (7.4%) developed an ECMO-related brain injury: 42 (5.3%) ischemic strokes, 20 (2.8%) intracranial bleeding and 3 diffuse microbleeds excluded from the analysis [12]. Among the 65 patients with ECMO-related brain injury, 2 patients had an ischemic stroke with a hemorrhagic transformation within their brain infarction and were classified as ischemic stroke patients as intracranial bleeding was secondary to the cerebral infarct. The annual incidence of stroke was stable over the study period. Clinical neurological symptoms leading to diagnosis are shown in Additional file 1: Table S1. Briefly, fixed dilated pupils, anisocoria, delayed awakening and proportional hemiplegia were the most frequent symptoms observed. Patients’ baseline characteristics according to brain-injury presence or absence are reported in Table 1. Reasons for ECMO support are reported in Additional file 1: Table S2, and patients’ outcomes in Table 2. Compared to patients with no damage or ischemic stroke, those with intracranial bleeding were more likely to be female, more frequently had central than peripheral VA-ECMO and more died. ECMO support before the neurological event was shorter for patients with intracranial bleeding than those with ischemic stroke. However, the latter were more often post-cardiac surgery patients than the others.

**Table 1:** Admission characteristics of VA-ECMO-treated patient according to neurological complication status

Characteristic	No damage (*n* = 813)	Brain injury	
		Ischemic stroke (*n* = 42)	Intracranial bleeding (*n* = 20)
Age (years)	53 [42;61]	50 [37;63]	44.5 [30;61]
Females^a^	237 (29)	12 (29)	11 (55)
Body mass index (kg/m^2^)	25 [23;28]	24 [22;29]	25 [22;31]
McCabe and Jackson comorbidity score ≥ 2	439 (54)	26 (62)	15 (75)
Previous history of stroke	44 (5)	0 (0)	3 (15)
At ICU admission			
SAPS II score	72 [55;85]	68.5 [60;86]	75.5 [55;91]
SOFA score	5 [3;14]	5 [3;8]	5 [3;9]
Organ failure			
Respiratory	530 (66)^b^	32 (76)	9 (47)
Hepatic	61 (8)	3 (7)	2 (10)
Renal	506 (63)	26 (62)	12 (60)
Hematological	377 (47)	28 (67)	12 (60)
Neurological	556 (69)	25 (60)	12 (60)
VA-ECMO hookup^a^			
Peripheral	705 (87)	29 (69)	11 (55)
Central	108 (13)	13 (31)	9 (45)
Cardiac surgery before or after ECMO^a^	329 (40)	25 (60)	8 (40)
Intra-aortic balloon pump	268 (33)	15 (36)	4 (20)
ENCOURAGE score^b^	23 [17;29]	21 [13;30]	25 [21;28]

Results are expressed as number (%) or median [27th;75th percentile]

*SAPS* Simplified Acute Physiology Score, *SOFA* sequential organ failure assessment, *VA-ECMO* venoarterial-extracorporeal membrane oxygenation

^a^*P *< 0.05 for between-group comparisons

^b^Calculated according to Muller et al. [2]

**Table 2:** Outcomes of VA-ECMO-treated patient according to neurological complication status

Characteristic	No damage (*n* = 813)	Brain injury	
		Ischemic stroke (*n* = 42)	Intracranial bleeding (*n* = 20)
At ECMO onset			
Gas-exchange value^b^			
pH	7.32 [7.17;7.42]	7.30 [7.21;7.42]	7.30 [7.15;7.41]
PaCO_2_	32.1 [26.2;38.9]	33.4 [27.4;37.9]	35.9 [26.3;42.4]
PaO_2_	160 [97;289]	144 [88;212]	240 [99;311]
Lactates	6 [2.7;11.1]	6 [2.4;10]	7.3 [4.6;11]
Platelet count (giga/L)^a,c^	155 [96;216]	165.5 [100;237]	80 [59;175]
Fibrinogen (g/L)	3 [2;5]	3.2 [2.4;4.8]	3 [1.7;4]
Prothrombin time (%)	45 [30;62]	52 [32;66]	30 [27;54]
aPTT, patient/normal-value ratio	1.6 [1.2;2.3]	1.5 [1.2;2.1]	1.9 [1.6;3.2]
ECMO duration before neurological event (days)^a^	–	11 [6;18]	5 [2;9]
ICU length of stay (days)^a^	11 [4;22]	18 [9;30]	5 [2;20]
Hospital mortality^a^	390 (48)	24 (57)	18 (90)
1-year mortality	NA	31 (73)	19 (95)

Results are expressed as number (%) or median [27th;75th percentile]

*VA-ECMO* venoarterial-extracorporeal membrane oxygenation, *ICU* intensive care unit, *aPTT* activated partial thrombin time, *NA* not available

^a^*P *< 0.05 for between-group comparisons

^b^Values are missing for 112 patients: 106 with no damage, 5 with ischemic stroke and 1 with intracranial bleeding

^c^Values are missing for 60 patients: 52 without no damage, 6 with ischemic stroke and 2 with intracranial bleeding

### Ischemic stroke patients

Specific univariable and multivariable analyses of the 42 ischemic stroke patients attempted to identify risk factors associated with this complication (Table 3). Multivariable analyses retained only central versus peripheral ECMO and platelets > 350 giga/L at ECMO start as being significantly associated with ischemic stroke, with respective OR [95% CI] of 3.2 [1.5–6.6] and 3.8 [1.4–10.7]. None of these factors were associated with hospital mortality (see Additional file 1: Table S5) or with the composite endpoint of death and ischemic stroke.

**Table 3:** Univariable and multivariable analyses of factors associated with ischemic stroke on VA-ECMO

Factor	Univariable analysis		Multivariable analysis	
	OR [95% CI]	*P* value	OR [95% CI]	*P* value
Age > 53 years	0.8 [0.4–1.5]	0.5		
Female sex	0.97 [0.5–1.9]	1		
SAPS II score at ICU admission ≥ 72	0.69 [0.4–1.3]	0.3		
Renal replacement therapy	1.6 [0.7–3.4]	0.4		
Intra-aortic balloon pump	1.13 [0.59–2.16]	0.7		
Central VA-ECMO	2.8 [1.4–5.5]	0.004	3.2 [1.5–6.6]	0.002
Post-cardiac surgery	2.2 [1.2–4.1]	0.01		
Blood tests at ECMO initiation				
Lactate > 6 mmol/L	1.0 [0.5–1.9]	1		
pH < 7.32	1.1 [0.6–2.1]	0.9		
Platelets > 350 giga/L	3.7 [1.3–10.0]	0.02	3.8 [1.4–10.7]	0.01
Bilirubin > 20 µmol/L	0.8 [0.4–1.5]	0.5		
Fibrinogen > 6 g/L	1.8 [0.7–5.0]	0.2		
Prothrombin time > 70%^a^	1.3 [0.5–3.5]	0.6		
aPTT, patient/normal-value ratio < 1.5	1.2 [0.6–2.4]	0.2		

*VA-ECMO* venoarterial-extracorporeal membrane oxygenation, *OR* odds ratio, *SAPS* Simplified Acute Physiology Score, *ICU* intensive care unit, *aPTT* activated partial thrombin time

^a^Expressed as a percentage of the normal value

**Fig. 2:**
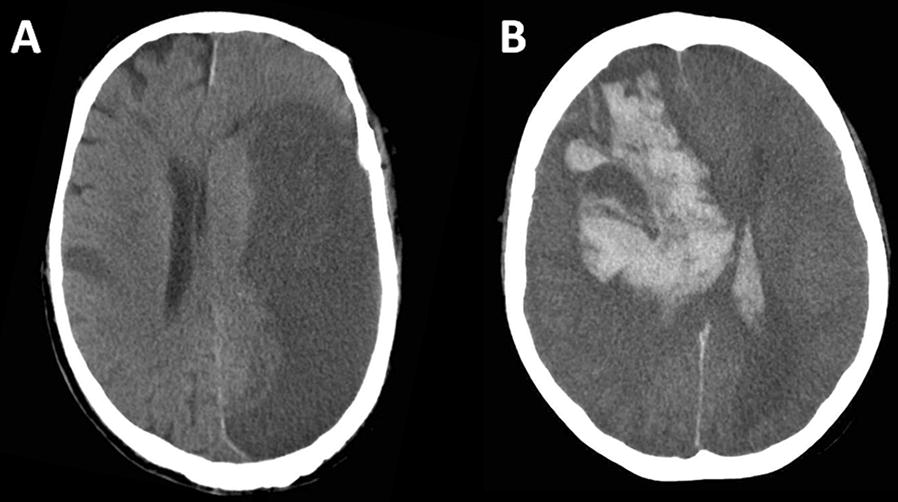
Brain CT-scan showing ECMO-related **A** massive cerebral infarction and **B** intraparenchymal hemorrhage

Because patient groups were heterogeneous, we designed a nested case–control study to evaluate potential associations of hemostasis parameters during ECMO support and ischemic stroke (Additional file 1: Table S3). Forty of our 42 cases could be matched with 86 controls, at least one per case. No relevant association could be established between anticoagulation use (with aPTT considered a surrogate marker of heparin dosage), fibrinogen level or platelet counts during ECMO and ischemic stroke. Figure 2A shows a brain CT-scan of an ECMO-related cerebral infarction.

### Patients with intracranial bleeding

Multivariable logistic-regression analysis of the risk factors of 20 patients with intracranial bleeding (Table 4) retained female sex, central versus peripheral VA-ECMO and platelets < 100 giga/L at ECMO onset as being significantly associated with intracranial bleeding, with respective OR [95% CI] of 2.9 [1.1–7.5], 3.8 [1.1–10.2] and 3.7 [1.4–9.7], and notably excluded hemostasis disorders during ECMO (particularly low platelet count). Whereas platelets < 100 giga/L at ECMO start were associated with death (see Additional file 1: Table S5) and with the composite endpoint of death plus intracranial bleeding (OR 1.7; 95% CI 1.2–2.3), female sex and central versus peripheral ECMO were not associated with these outcomes.

**Table 4:** Univariable and multivariable analyses of factors associated with intracranial bleeding on VA-ECMO

Factor	Univariable analysis		Multivariable analysis	
	OR [95% CI]	*P* value	OR [95% CI]	*P* value
Age > 53 years	0.6 [0.2–1.5]	0.3		
Female sex	3 [1.2–7.3]	0.02	2.9 [1.1–7.5]	0.03
Previous history of stroke	3 [0.9-10.92]	0.1		
SAPS II at ICU admission ≥ 72	1.2 [0.5–2.8]	0.8		
Renal replacement therapy	2 [0.6–7]	0.3		
Intra-aortic balloon pump	0.5 [0.2–1.5]	0.3		
Central VA-ECMO	5.0 [2.0–12.2]	0.0007	3.8 [1.1–10.2]	0.008
Cardiac surgery	0.9 [0.4–2.3]	1		
Biology at ECMO onset				
Lactate > 6 mmol/L	2.7 [0.9–7.6]	0.06		
pH < 7.32	1.0 [0.4–2.6]	1		
Platelets < 100 giga/L	4.3 [1.7–11.3]	0.003	3.7 [1.4–9.7]	0.009
Bilirubin > 33 µmol/L	1.8 [0.7–4.8]	0.3		
Fibrinogen < 1.5 g/L	1.7 [0.5–6.2]	0.4		
Prothrombin time < 30%^a^	2.7 [1.0–7.7]	0.07		
aPTT, patient/normal-value ratio > 3	2.3 [0.8–6.7]	0.2		
Hemostasis disorders on ECMO^b^				
Platelets < 100 × giga/L	1.2 [0.4–4.3]	1		
Prothrombin time < 30%^a^	2.0 [0.8–5.0]	0.1		
Fibrinogen < 1.5 g/L	1.9 [0.7–4.9]	0.2		
aPTT, patient/normal-value ratio > 3	1.1 [0.4–3.1]	0.8		

*VA-ECMO* venoarterial-extracorporeal membrane oxygenation, *OR* odds ratio, *SAPS* Simplified Acute Physiology Score, *ICU* intensive care unit, *APTT* activated partial thrombin time

^a^Expressed as a percentage of the normal value

^b^Worst value on ECMO and before intracranial bleeding

Because it was been previously shown that blood-gas change at ECMO onset is associated with intracranial bleeding in patients receiving VV-ECMO [11], a nested case–control study was undertaken to evaluate this association in VA-ECMO-treated patients (see Additional file 1). Nineteen of our 20 cases with intracranial bleeding could be matched with 58 controls. Cases’ PaCO_2_ decreased more after ECMO onset, and they had lower platelet counts at ECMO start compared to the controls (Additional file 1: Table S4). No pattern of a specific location or aspect of ECMO-related brain hemorrhage was identified among all brain imaging, and within the 20 patients with intracranial bleeding, we identified, 13 patients (65%) with intraparenchymal hemorrhage (among which 4 patients with multiples lesions), 4 patients (20%) with subarachnoid hemorrhage and 3 patients (15%) with acute subdural hematoma. Figure 2B shows a brain CT-scan of an ECMO-related intraparenchymal hemorrhage.

## Discussion

Herein, we described one of the largest and most detailed populations of VA-ECMO–treated patients evaluated for neurological complications. Neurological events were common, occurring in 7.4% of patients. Ischemic stroke was the most frequent, representing 65% of events (5.3% of our VA-ECMO-treated population). It occurred mainly during the second week of ECMO support and was not associated with higher mortality, compared to patients with no damage. Platelets > 350 giga/L at ECMO initiation were the only risk factor identified for this ischemic complication. Intracranial bleeding occurred more rarely, affecting 2.8% of patients, and earlier (during the first week of ECMO support for most patients), and was associated with high mortality, female sex, central ECMO and low platelet count. Moreover, our nested case–control study results suggested that, as for VV-ECMO patients, rapid correction of PaCO_2_ at ECMO start seemed to be associated with intracranial bleeding [11].

Brain injury is a well-known complication of VA- and VV-ECMO support. Brain-injury frequency and risk factors on VV-ECMO were recently investigated [11, 15]. Larger VA-ECMO series exist. Recently, Nasr et al. reviewed 23,951 patients from the Nationwide Inpatient Sample and found 4.1% ischemic stroke and 3.6% intracranial bleeding [5]. Although that study included a large number of patients, they assessed VV- and VA-ECMO together, and risk factors for neurological complications were not examined. However, as in our study, they found that intracranial bleeding, but not ischemic stroke, was associated with higher mortality. In their analysis of 2000 ECMO runs in a single hospital, Gray et al. found that adults treated with ECMO for acute heart failure had 8% ischemic stroke and intracranial bleeding with a 43% survival rate [16]. That study too mixed ischemic stroke and intracranial bleeding and did not evaluate risk factors for brain injury. Lorusso et al. evaluated 4522 patients included in the Extracorporeal Life Support Organization registry [7]; 15.1% neurological complications occurred during VA-ECMO, and analysis identified pre-ECMO cardiac arrest, inotrope use on ECMO and hypoglycemia as risk factors for brain injury. However, neurological complication was defined as brain death, seizures, cerebral infarction or cerebral hemorrhage and all complications were regrouped together in their analysis. Moreover, those authors did not specifically examine risk factors for each complication. Nevertheless, the incidences of ischemic and hemorrhagic stroke in Lorusso study were, respectively, at 3.6% and 1.8% [7], whereas in our study, they were, respectively, at 5.3% and 2.8%. This small higher incidence in our work could be explained by case-mix differences: Lorusso et al. reported less cardiomyopathy and cardiac surgery compared to our population, conditions associated with higher rates of ECMO-related brain injury in our study.

We were unable to establish any relevant risk factor for ischemic stroke, even with our nested case–control study; neither hemostasis parameters nor anticoagulation use was associated with it. Although disappointing, this finding is not surprising. ECMO is not the only risk factor for ischemic events in these patients; cardiac surgery, myocardial infarction, cardiac arrest, low cerebral output flow consecutive to heart failure or other conditions may also be responsible. Thus, it is difficult to know the respective impact of underlying disease(s) and ECMO itself. However, ECMO might play a role, because of this population’s high rate of unrecognized systemic thromboembolic events [17]. In a small monocenter study, Omar et al. evaluated risk factors in a mixed VA- or VV-ECMO population, among whom 5.8% experienced an ischemic stroke; their multivariable analysis retained pre-ECMO blood lactates > 10 mmol/L as being independently associated [18]. Multiple factors probably contribute to ischemic stroke, with disease severity at ECMO onset being one of them. Transcranial Doppler monitoring might be informative, because a recent preliminary study showed that it detected microembolic signals in nine out of 11 VA-ECMO patients [19]. Although that study included only a small number of patients and the authors did not observe any neurological complications, their observations deserve further investigation on a larger scale.

Few studies have sought risk factors for intracranial bleeding in VA-ECMO patients. Kasirajan et al. [20] studied 78 VA-ECMO-treated patients, among whom 18.9% had intracranial bleeding. They found that intracranial bleeding was associated with female sex, renal failure (and dialysis) and thrombocytopenia. Although that study was performed 18 years ago and used a different ECMO circuit, we also found intracranial bleeding to be associated with female sex or low platelets at ECMO onset, and similar high mortality. More recently, Sandersjöö et al. [21] reported cerebral bleeding in 21% of 253 ECMO-supported patients (161 VV-ECMO, 92 VA-ECMO) and that pre-ECMO antithrombotic treatment and low platelets on ECMO were associated with this complication. However, that study, too, mixed VV- and VA-ECMO patients, and although the pathophysiology of bleeding might be similar for both ECMO hookups, several technical differences (e.g., increased venous pressure in the jugular cannula may play a role but is specific to VV-ECMO patients) might explain the different results obtained in their study and ours. Indeed, most risk factors of our patients with intracranial bleeding were seen before or just after starting ECMO: low platelets and rapid PaCO_2_ change at ECMO implantation. Although it is impossible to precisely determine the intracranial bleeding mechanism in those patients, it is highly probable that it is a multifactorial process with multiple events that may injure the brain and its vessels leading to bleeding, with hemostasis disorders before and at ECMO start and rapid PaCO_2_ change being among them. Sandersjöö et al. [21] found similar associations between cerebral bleeding (in VV- and VA-ECMO patients) and pre-ECMO parameters, e.g., preadmission antithrombotic therapy, high pre-cannulation SOFA coagulation score and septic shock. These findings warrant further investigation to understand the precise mechanisms of this injury.

As shown in our study, platelet count < 100 giga/L at ECMO onset is a risk factor for intracranial bleeding. We therefore recommend to keep a platelet count up to 100 giga/L during VA-ECMO insertion, and to infuse low dose heparin (except in case of bleeding) to avoid circuit clotting, that may induce by itself thrombopenia. However, since platelet count < 100 giga/L at ECMO onset is also associated with death or the composite endpoint of death plus intracranial bleeding, we cannot exclude that this may be associated with death rather than with intracranial bleeding. We also found that platelets level > 350 giga/L at ECMO onset is a risk factor for ischemic stroke. However, we do not recommend adding systematically antiplatelet agents, except in patients requiring these agents, based on medical history.

The main metabolic factors known to cause significant changes in cerebral blood flow are PaCO_2_ and pH, due to vascular smooth muscle cells vasoconstriction. These parameters can rapidly change during ECMO. Intracranial bleeding is the most frequent cerebrovascular complication during VV-ECMO, and it has been shown that a too rapid PaCO_2_ decrease after ECMO start was independently associated with this complication [11]. However, because rapid decrease in PaCO_2_ leads to cerebral vasoconstriction, the relationship between PaCO_2_ change and cerebral bleeding is difficult to understand, because vasoconstriction should lead to ischemic strokes. One hypothesis could be that those cerebral bleeding where in fact secondary hemorrhagic transformation after cerebral infarction due to a cerebral vasoconstriction induced by ECMO. Another hypothesis is a multiple hits model, in which rapid PaCO_2_ change at ECMO start is only one of the hit and that other conditions (before or after ECMO start) are needed to trigger intracranial bleeding. Whether or not any deleterious effect may occur on endothelial cells remains to be determined.

### Study limitations

Our study has several limitations. The first is its retrospective, single-center design. However, this large study covered a long period and analyzed previously unexamined risk factors for ischemic stroke and intracranial bleeding. Therefore, we think our results may be transposable to other ICUs with other patients. Second, because patients with clinical symptoms underwent brain imaging, it is highly likely that we may have missed some subclinical events. Indeed, Rastan et al. [17] identified during autopsy a high percentage of patients with thromboembolic events that were not clinically apparent, and a recent study on VV-ECMO-treated patients showed that systematic cerebral computed-tomography scans may find clinically unapparent bleeding events [22]. Third, we tried to find risk factors associated with ischemic stroke and intracranial bleeding, but other potentially confounding factors may not have been considered in our analyses. More specifically, we examined some, but not all, hemostasis parameters known to be modified during ECMO as risk factors for intracranial bleeding, e.g., von Willebrand factor [23]. Thus, our conclusion that hemostasis disorders during ECMO were not involved in neurological events (mainly intracranial bleeding) might be inaccurate. In addition, the potential role of laminar versus pulsed flow in the pathogenesis of neurological complications, mainly ischemic stroke, was not considered. Indeed, some patients may have residual left ventricular ejection on VA-ECMO that might be implicated, especially in ischemic stroke. Unfortunately, because of our study’s retrospective design, this information is not available. Fourth, some variables included in the analyses are not baseline characteristics and may only identify the sickest VA-ECMO patients rather than true risk factors. Fifth, we have no data on long-term quality of life. Although mortality was not different between patients without brain injury and those experiencing ischemic stroke, differences in quality of life may exist. This should be explored in future studies. Sixth, we were unable to have the number of patients who underwent cerebral imaging because of abnormal neurological findings and whose imaging was normal. Last, we decided to exclude patients who developed neurological complication 7 days after ECMO removal, which could underestimate the incidence of brain injury. We hypothesized that a neurological complications occurring more than 7 days after ECMO removal had a low probability to be directly related to the ECMO itself. Since it is our policy to stop sedation after ECMO removal for neurological evaluation, it is highly probable that we are able to diagnose brain injury in the first week after ECMO removal, if this injury occurs during ECMO course. Indeed, there are many reasons for these patients to develop brain injury after ECMO removal: emboli from a failing heart, anticoagulation, etc. Moreover, median time from ECMO removal to diagnosis of brain injury in the patients who were excluded was 13 (IQR 11–21) days, reinforcing our hypothesis.

## Conclusions

Structural brain injuries are common in VA-ECMO-treated patients. Ischemic stroke seems to be the most frequent and occurs after the first week of ECMO support, but without specific risk factors except platelets > 350 giga/L at ECMO start. Ischemic stroke does not seem to be associated with higher mortality, compared to patients with no damage. Intracranial bleeding is less frequent but occurs earlier, during the first week on ECMO, and is associated with female sex, central ECMO (vs. peripheral) and low platelets at ECMO start, and higher mortality. Moreover, as for VV-ECMO [11], rapid PaCO_2_ change at ECMO onset also seems to be associated with enhanced risk of intracranial bleeding. Whether therapeutic interventions (platelet transfusion or slow and progressive PaCO_2_ correction) might decrease this fatal complication remains to be determined.

## Additional file

**Additional file 1:** **Table S1.** Cerebral imaging findings and their corresponding clinical features. **Table S2.** Reason for VA-ECMO according to neurological complication status. **Table S3.** Characteristics and hemostasis parameters of the VA-ECMO–treated patients included in the nested case–control study for ischemic stroke risk-factor analysis. **Table S4.** Characteristics, hemostasis parameters, blood-gas values and changes for the VA-ECMO–treated patients included in the case–control study for intracranial bleeding risk-factor analysis. **Table S5.** Univariable and multivariable analysis of factors associated with hospital mortality.

## Abbreviations

aPTT: activated partial thrombin
ECMO: extracorporeal membrane oxygenation
ICU: intensive care unit
SAPS: Simplified Acute Physiology Score
SD: standard deviation
VA: venoarterial
VV: venovenous
