# Supplementary material for: Ischemic and hemorrhagic brain injury during venoarterial-extracorporeal membrane oxygenation
Source: Ann Intensive Care. 2018 Dec 20;8:129. doi: 10.1186/s13613-018-0475-6 (PMC6301905; doi:10.1186/s13613-018-0475-6)
Supplement: Supplementary file 1 — Additional file 1: Table S1. Cerebral imaging findings and their corresponding clinical features. Table S2. Reason for VA-ECMO according to neurological complication status. Table S3. Characteristics and hemostasis parameters of the VA-ECMO–treated patients included in the nested case–control study for ischemic stroke risk-factor analysis. Table S4. Characteristics, hemostasis parameters, blood-gas values and changes for the VA-ECMO–treated patients included in the case–control study for intracranial bleeding risk-factor analysis. Table S5. Univariable and multivariable analysis of factors associated with hospital mortality. [file 13613_2018_475_MOESM1_ESM.docx]

**Ischemic and hemorrhagic brain injury during venoarterial-extracorporeal membrane oxygenation**

Loïc Le Guennec^,^ Clémentine Cholet, Florent Huang, Matthieu Schmidt, Nicolas Bréchot, Guillaume Hékimian, Sébastien Besset, Guillaume Lebreton, Ania Nieszkowska, Pascal Leprince, Alain Combes and Charles-Edouard Luyt

**Online supplement**

**Methods**

**Anticoagulation management during ECMO**

All patients received the same anticoagulation protocol as that described previously [11, 13, 14]. After a heparin bolus (5000 IU) at ECMO initiation, all patients were continuously infused with unfractionated heparin. The heparin dose was adapted, at least once daily, according to the activated partial thrombin time (aPTT) (expressed as patient/normal-value ratio, targeting 1.5–2-fold the normal-control value) and clinical tolerance; heparin was stopped when bleeding occurred and restarted once it was controlled. Bleeding requiring heparin withdrawal was defined as any clinical bleeding (at the ECMO implantation site, central or arterial lines, tracheal secretions, ear nose and throat), with or without hemodynamic impact or hemoglobin decline, judged meaningful by the patient’s treating physician. Anticoagulant overdose was defined biologically as an aPTT >2.5, corresponding to an absolute value of ≥80 s.

The membrane oxygenator and its circuitry were checked daily by experienced perfusionists and changed when: fibrin deposition or thrombi had deleterious effects on blood oxygenation; platelet count (<20 giga/L) or blood fibrinogen (<1.5 g/L) decreased markedly; or intravascular hemolysis (twice-measured free plasma hemoglobin >200 mg/L and no other cause of mechanical hemolysis) was found. No systematic circuit change was scheduled.

**Nested Case–control studies**

To compare specific risk factors for intracranial bleeding and ischemic stroke, we designed 2 nested case–control studies.

The first compared patients with ischemic stroke to those with no brain damage (controls), matched for sex, age ± 5 years, SAPS II ± 5, ENCOURAGE mortality-risk score ± 5, ECMO duration at least equal to that of ischemic stroke onset for the paired cases, and, if possible, reason for ECMO. For each case with more than one possible control, up to three controls were selected. Then hemostasis parameters for the two groups were compared. Assuming that ischemic stroke patients could have received less anticoagulation and could have more “supranormal” hemostasis parameters than those without (i.e., highest platelet counts and fibrinogen levels), we evaluated platelet counts, fibrinogen level and activated partial thrombin time (aPTT) as markers of anticoagulation. For those three parameters, we examined their highest and lowest values during ECMO, as well as the number of days on which their values were comprised within several ranges.

The second nested case–control study compared patients with intracranial bleeding and those with no brain damage (controls) matched for baseline criteria for the controls were age ± 5 years, sex, SAPS II score ± 5, ENCOURAGE mortality-risk score ± 5, ECMO duration at least equal to that of intracranial bleeding onset for the paired cases, and, if possible, reason for ECMO. For each case having more than one possible control, up to three controls were selected. Then, the new matched-data set was evaluated to assess the role of blood-gas changes and hemostasis disorders on brain injury.

**Statistical analysis**

All these analyses used methods that accounted for the matched design. Thus, continuous variables were compared using a matched *t*-test and dichotomous outcomes were compared using the McNemar test. Analyses were computed with StatView v5.0 (SAS Institute Inc, Cary, NC) and SPSS v11.5 (SPSS Inc, Chicago, IL) software. *P* < 0.05 defined significance.

**Results**

**Case–control study**

*Patients with ischemic stroke*

As shown in *Table S3*, 40 of our 42 cases could be matched to 86 controls, with each case having at least one matched control. Baseline characteristics of cases and controls were similar, and hemostasis parameters differed only for the number of days with low (<150 giga/L) platelets, which was significantly higher for controls, and the number of days with aPTT >2, which was significantly higher for cases.

*Patients with intracranial bleeding*

As shown in *Table S4*, 19 of our 20 patients with intracranial bleeding could be matched to 57 controls. Baseline characteristics of cases and controls were similar except for age, with controls being slightly older. Intracranial bleeding patients, compared to their matched controls, had significantly higher pH increases and PaCO_2_ decreases just after ECMO onset. As for the whole-population analysis, intracranial bleeding patients had significantly lower platelet counts at ECMO implantation, whereas their hemostasis parameters during ECMO did not differ from those of patients with no damage. Notably, compared to patients without, patients with intracranial bleeding tended to have lower fibrinogen levels, shorter prothrombin time and higher aPTT at ECMO implantation.

**Table S1** **Cerebral imaging findings and their corresponding clinical features**

| **Clinical Picture** | **Ischemic stroke (*n* = 42)**  **Number of patients (%)** | **Intracranial bleeding (*n* = 20)**  **Number of patients (%)** |
| --- | --- | --- |
| Fixed dilated pupils | 2 (4) | 10 (50) |
| Anisocoria | 5 (11) | 7 (35) |
| Delayed awakening | 13 (30) | 2 (1) |
| Seizures | 2 (4) | 2 (1) |
| Confusion | 1 (2) | 0 (0) |
| Facial paralysis | 13 (30) | 1 (0.5) |
| Hemiparesis | 4 (10) | 0 (0) |
| Proportional hemiplegia | 14 (33) | 0 (0) |
| Aphasia | 5 (11) | 0 (0) |

**Table S2. Reason for VA-ECMO according to neurological complication status**

| **Reason for ECMO** | **No damage**  **(*n* = 813)** | **Brain injury** | |
| --- | --- | --- | --- |
|  |  | **Ischemic stroke**  **(*n* = 42)** | **Intracranial bleeding**  **(*n* =20)** |
| Cardiac arrest | 156 (19) | 5 (12) | 2 (10) |
| Septic shock | 59 (7) | 3 (7) | 2 (10) |
| Chronic dilated cardiomyopathy | 140 (17) | 7 (17) | 4 (20) |
| Pulmonary embolism | 6 (1) | 0 | 1 (5) |
| Endocarditis | 8 (1) | 1 (2) | 0 |
| Myocardial infarction | 124 (15) | 4 (10) | 2 (10) |
| Drug intoxication | 9 (1) | 0 | 0 |
| Myocarditis | 47 (6) | 3 (7) | 0 |
| Electrical storm | 9 (1) | 0 | 0 |
| Post-cardiac surgery | 221 (27) | 17 (40) | 6 (30) |
| Cardiac graft rejection | 11 (1) | 0 | 0 |
| ARDS with cardiogenic shock | 17 (2) | 1 (2) | 2 (10) |
| Miscellaneous | 5 (1) | 0 | 0 |

Results are expressed as number (%). Abbreviations: VA-ECMO, venoarterial-extracorporeal membrane oxygenation; ARDS, acute respiratory distress syndrome.

**Table S3** **Characteristics and hemostasis parameters of the VA-ECMO–treated patients included in the nested case–control study for ischemic stroke risk-factor analysis**

| **Characteristic** | **Cases**  **(*n* = 40)** | **Controls**  **(*n* = 86)** |
| --- | --- | --- |
| Age, years | 51 [40;63] | 51 [37;60] |
| Females | 13 (33) | 29 (34) |
| ENCOURAGE score^a^ | 24 [13;30] | 24 [16;29] |
| SAPS II | 70 [52;83] | 64 [54;81] |
| Reason for VA-ECMO |  |  |
| Cardiac arrest | 2 (5) | 4 (5) |
| Septic shock | 5 (13) | 9 (10) |
| Chronic dilated cardiomyopathy | 7 (18) | 19 (22) |
| Myocardial infarction | 7 (18) | 17 (20) |
| Post-cardiac surgery | 12 (30) | 24 (28) |
| Myocarditis | 4 (10) | 7 (8) |
| Cardiac transplant rejection | 0 | 0 |
| Others | 3 (8) | 2 (2) |
| Hemostasis during VA-ECMO |  |  |
| Highest platelet counts, giga/L | 202 [132;316] | 182 [131;277] |
| Lowest platelet counts, giga/L | 34 [24;55] | 31 [18;53] |
| No. of days with platelets <150 giga/L^b^ | 8 [5;10] | 10 [6;17] |
| No. of days with platelets ≥150 and ≤500 giga/L | 0 [0;1] | 0 [0;1] |
| No. of days with platelets >500 giga/L | 0 [0;0] | 0 [0;0] |
| Highest aPTT^c^ | 2.9 [1.9;3.5] | 3.0 [2.2;4.2] |
| Lowest aPTT^c^ | 1.1 [1;1.2] | 1.1 [1.0;1.3] |
| No. of days with aPTT ≥1 and ≤1.1^c^ | 1 [0;3] | 0 [0;18] |
| No. of days with aPTT ≥1.11 and ≤1.20^c^ | 1 [0;3] | 1 [0;2] |
| No. of days with aPTT ≥1.21 and ≤1.50^c^ | 4 [3;8] | 4 [2;9] |
| No. of days with aPTT ≥1.51 and ≤2.0^b,c^ | 2 [1;4] | 3 [1;7] |
| No. of days with aPTT >2^b,c^ | 2 [0;2] | 1 [0;4] |
| Highest fibrinogen level, g/L | 6.6 [4.7;7.9] | 6.5 [4.6;7.7] |
| Lowest fibrinogen level, g/L | 1.9 [1.4;3.2] | 1.8 [1.3;2.6] |
| No. of days with fibrinogen level <2 g/L | 0 [0;1] | 0 [0;1] |
| No. of days with fibrinogen level >3.5 but ≤6 g/L | 6 [2;11] | 6 [2;12] |
| Number of days with fibrinogen level >6 g/L | 2 [0;6] | 1 [0;5] |

Results are expressed as n (%) or median [25^th^;75th percentile].

Abbreviations: SAPS, Simplified Acute Physiology Score; VA-ECMO, venoarterial-extracorporeal membrane oxygenation; aPTT, activated partial thrombin time.

^a^ Calculated from Muller *et al*. (2)

^b^*P* < 0.05.

^c^Expressed as the patient/normal-control value ratio.

**Table S4 Characteristics, hemostasis parameters, blood-gas values and changes for the VA-ECMO–treated patients included in the case–control study for intracranial bleeding risk-factor analysis**

| **Characteristic** | **Cases**  **(n = 19)** | **Controls**  **(n = 57)** |
| --- | --- | --- |
| Age, years | 42 [29;57] | 48 [33;59] |
| Females | 8 (47) | 31 (53) |
| ENCOURAGE score^a^ | 25 [21;28] | 24 [17;28] |
| SAPS II | 74 [53;86] | 73 [56;86] |
| Reason for VA-ECMO |  |  |
| Cardiac arrest | 2 (12) | 5 (9) |
| Septic shock | 4 (24) | 8 (14) |
| Chronic dilated cardiomyopathy | 4 (24) | 17 (29) |
| Myocardial infarction | 3 (18) | 10 (17) |
| Post-cardiac surgery | 4 (24) | 17 (29) |
| Myocarditis | 0 | 2 (3) |
| Cardiac transplant rejection | 0 | 2 (3) |
| Others | 0 | 1 (2) |
| Hemostasis parameters at ECMO start |  |  |
| Platelets, giga/L^b^ | 80 [53;160] | 144 [76;229] |
| Prothrombin time, % | 28 [26;49] | 46 [27;66] |
| Fibrinogen, g/L | 2.5 [1.4;4] | 3.0 [2.0;4.8] |
| aPTT, patient-to-normal value ratio | 2.1 [1.6;3.4] | 1.6 [1.3;2.6] |
| Hemostasis parameters on ECMO^c^ |  |  |
| Platelets, giga/L | 29 [16;90] | 31 [20;54] |
| Prothrombin time, % | 33 [24;52] | 34 [22;52] |
| Fibrinogen, g/L | 2.6 [1.5;3.9] | 2.3 [1.4;3.4] |
| aPTT, patient-to-normal value ratio | 3.2 [1.9;4.4] | 3.1 [2.1;4.3] |
| Gas-exchange values pre-ECMO |  |  |
| pH^b^ | 7.23 [7.12;7.34] | 7.36 [7.25;7.42] |
| PaO_2_, mmHg | 111 [82;135] | 126 [98;196] |
| PaCO_2_, mmHg^b^ | 42 [98;46] | 31 [25;37] |
| Bicarbonates, mmol/L | 16 [14;19] | 16 [11;22] |
| Lactates, mmol/L | 6.3 [2.9;10.4] | 6.2 [2.2;12.2] |
| Gas-exchange values post-ECMO |  |  |
| pH | 7.41 [7.18;7.49] | 7.33 [7.24;7.41] |
| PaO_2_, mmHg | 262 [181;337] | 271 [182;336] |
| PaCO_2_, mmHg | 26 [20;32] | 29 [22;33] |
| Bicarbonates, mmol/L | 15 [1;20] | 15 [10;19] |
| Lactates, mmol/L | 9.1 [4.8;11.9] | 7.7 [4.2;12.3] |
| Gas-exchange–value changes^d^ |  |  |
| pH change^b^ | 0.14 [0.05;0.25] | ;0.01 [–0.1;0.07 ] |
| PaO_2_ change | 139 [5;173] | 99 [38;182] |
| PaCO_2_ change^b^ | –13.3 [–23.7;–3.3] | –0.6 [–6.5;5] |

Results are expressed as n (%) or median [25^th^;75th percentile].

Abbreviations: SAPS, Simplified Acute Physiology Score; VA-ECMO, venoarterial-extracorporeal membrane oxygenation.

^a^Calculated from Muller *et al*. (2)

^b^*P* < 0.05.

^c^Worst value on VA-ECMO and before intracranial bleeding.

^d^Defined as the post-ECMO pH, PaCO_2_ or PaO_2_ value minus the pre-ECMO pH, PaCO_2_ or PaO_2_ value.

Table S5: Univariable and multivariable analysis of factors associated with hospital mortality

|  | Univariable analysis | |  | Multivariable analysis | |
| --- | --- | --- | --- | --- | --- |
|  | OR (95% CI) | P value |  | OR (95% CI) | P value |
| Age >53 years | 1.8 (1.4–2.3) | <0.0001 |  | 2.1 (1.5–3.0) | <0.0001 |
| Female sex | 1.1 (0.8–1.5) | 0.4 |  |  |  |
| SAPS II score at ICU admission ≥72 | 3.5 (2.7–4.7) | <0.0001 |  | 1.9 (1.3–2.8) | <0.0001 |
| Renal replacement therapy | 4.9 (3.4–6.9) | <0.0001 |  | 3.8 (2.4–5.9) | 0.0006 |
| Intra-aortic balloon pump | 0.9 (0.6–1.1) | 0.3 |  |  |  |
| Central VA-ECMO | 0.9 (0.6–1.2) | 0.4 |  |  |  |
| Post cardiac surgery | 0.4 (0.3–0.5) | <0.0001 |  | 0.5 (0.3–0.7) | 0.0001 |
| Organ failure at ECMO start  Lung  Liver  Brain  Kidney | 1.3 (1.0–1.8)  1.7 (1.02–2.8)  2.6 (1.9–3.5)  2.4 (1.8–3.2) | 0.04  0.04  <0.0001  <0.0001 |  | 2.7 (1.3–5.9) | 0.01 |
| Blood tests at ECMO initiation |  |  |  |  |  |
| Lactate >6 mmol/L | 3.1 (2.3–4.2) | <0.0001 |  | 1.7 (1.2–2.5) | 0.005 |
| pH <7.32 | 2.2 (1.6–2.9) | <0.0001 |  |  |  |
| Platelets <100 giga/L | 1.7 (1.2–2.3) | 0.001 |  | 1.8 (1.2–2.7) | 0.005 |
| Platelets >350 giga/L | 0.7 (0.4–1.4) | 0.4 |  |  |  |
| Bilirubin >33 µmol/L | 1.0 (0.7–1.4) | 0.9 |  |  |  |

Abbreviations: VA-ECMO, venoarterial-extracorporeal membrane oxygenation; OR, Odds ratio; SAPS, Simplified Acute Physiology Score; ICU, intensive care unit; APTT, activated partial thrombin time.
